# Administration with carnosic acid alleviates the development of osteoarthritis by attenuating macrophage polarization-mediated inflammation and cartilage oxidative damage and degradation via regulating Nrf2/NF-kB axis

**DOI:** 10.3389/fimmu.2026.1710302

**Published:** 2026-02-10

**Authors:** Yang Yang, Wenwen Ping, Jin Xu, Rong Yang

**Affiliations:** 1 Department of Cardiology, The First Affiliated Hospital of Nanjing Medical University, Nanjing, China; 2 Department of Rheumatology, Zhongda Hospital, School of Medicine, Southeast University, Nanjing, China

**Keywords:** carnosic acid, inflammation, macrophage polarization, osteoarthritis, oxidative stress

## Abstract

**Introduction:**

Osteoarthritis (OA) is a common inflammatory degenerative joint disease characterized by deterioration of articular cartilage. Macrophages exert the important roles in cartilage damage and synovial inflammation in OA. Carnosic acid (CA) possesses critical function in multiple inflammatory diseases.

**Methods:**

Macrophages were treated with carnosic acid. Chondrocytes were incubated with conditioned medium (CM) from macrophages. Then, the effects on macrophage polarization were analyzed. The effects on chondrocyte oxidative injury, inflammation and extracellular matrix (ECM) metabolism imbalance were explored. Carnosic acid was administrated to Anterior cruciate ligament transection (ACLT)-constructed OA mice model. Then, the efficacy of carnosic acid in the progression of OA was further explored.

**Results:**

Carnosic acid inhibited LPS-induced macrophage M1 polarization and enhanced IL-4-evoked macrophage M2-like polarization. Furthermore, conditioned medium (CM) from M1 macrophages induced chondrocyte oxidative injury, which were abrogated by carnosic acid. Concomitantly, carnosic acid restrained M1 macrophage CM-increased inflammatory mediator and inflammatory cytokine IL-6 and TNF-α contents. Moreover, carnosic acid also antagonized M1 macrophage CM-evoked extracellular matrix (ECM) degradation. Mechanistically, carnosic acid enhanced Nrf2 expression and suppressed subsequent activation of the NF-κB signaling. Intriguingly, Nrf2 inhibition restrained activation of the NF-κB signaling and reversed carnosic acid-mediated suppression on macrophage M1-like polarization. Furthermore, carnosic acid attenuated M1 macrophage-induced oxidative injury, inflammatory response and ECM metabolism disturbance of chondrocytes. *In vivo*, carnosic acid alleviated articular cartilage degeneration and synovitis, leading to decrease of Osteoarthritis Research Society International (OARSI) and synovitis scores. Moreover, carnosic acid attenuated oxidative stress injury and cartilage degradation in OA mice. Additionally, carnosic acid affected synovium macrophage polarization from M1 to M2 , mitigated inflammation and activation of the Nrf2/NF-kB axis in OA mice.

**Discussion:**

Carnosic acid may alleviate the progression of OA by attenuating macrophage polarization-mediated inflammatory response and cartilage oxidative damage via regulating Nrf2/NF-kB axis, supporting it as a promising and potential therapeutic agent for patients with OA in clinical practice.

## 1 Introduction

Osteoarthritis (OA) is a chronic and progressive degenerative joint disease, and the epidemiological data confirm the increased prevalent cases of OA from 247.51 million in 1990 to 527.81 million in 2019 (1, 2). OA is a debilitative and painful disease, and represents the 11^th^ leading cause of disability worldwide (3). Currently, OA has become a major public health concern worldwide and greatly affects quality of life and life expectancy in ageing people. Moreover, the presence of OA is often accompanied by other common chronic conditions in older people, which may increase the risk of adverse outcomes (3, 4). Notably, OA imposes a heavy societal burden, with OA-related medical costs increase surging from €8.6 billion in 2015 to €12.1 billion in 2020, particularly among older adults in Germany (5). Although remarkable progress has been achieved in OA, the current pharmacological agents (such as non-steroidal anti-inflammatory drugs) and the conventional joint replacement surgery for the advanced OA patients have the limited efficacy due to the undesirable adverse effects and postoperative complications (6). Thus, exploring new therapeutic strategy is imperative for OA management.

As a chronic inflammatory and degenerative joint disease, OA is usually characterized by cartilage degeneration and synovial inflammation. The current pathological mechanisms underlying OA are complicated, and a growing body of research has implicated oxidative stress and inflammatory response in the pathogenesis of OA (7–10). Oxidative stress refers to a well-established imbalance between the antioxidant defense system and the generation of reactive oxygen species (ROS). Intriguingly, the previous studies revealed high oxidative levels in the articular cartilage of OA (8, 9). Moreover, excessive oxidative stress may induce chondrocyte apoptosis and lead to articular cartilage destruction and degeneration by increasing extracellular matrix (ECM) catabolic enzyme matrix metalloproteinases (MMPs) and decreasing ECM synthesis (9). Currently, synovial inflammation is a key contributor for OA and is closely correlated with disease severity, such as deterioration of cartilage degeneration, pain and joint symptoms (7). Macrophages constitute the major immune cells within the synovium, and has been implicated in the pathological progression of OA. Generally, macrophages typically polarize into two distinct phenotypes under various stimuli: the classically pro-inflammatory M1 phenotype and the anti-inflammatory M2 phenotype. Notably, the inflammatory microenvironment in OA promotes the polarization of synovial macrophages toward M1-like phenotype, which will secrete abundant pro-inflammatory mediators and cytokines to aggravate the progression of OA, such as IL-1β and IL-6 (7, 11). A previous report confirmed a direct association between the imbalance of M1/M2 macrophage and OA severity (12). Currently, lowering oxidative stress and repolarizing macrophages-mediated inflammation represent a promising approach for the treatment of OA (11, 13, 14).

Carnosic acid is a natural phenolic diterpene and bioactive compound originally isolated from rosemary (*Rosmarinus officinalis*), and has been corroborated to exert multiple beneficial efficiencies in inflammation-related diseases and oxidative injury (15–17). For instance, emerging evidence supports naturally-occurring carnosic acid as a promising therapeutic agent for skin inflammation (16). Moreover, carnosic acid can ameliorate indomethacin-induced gastric ulceration by inhibiting oxidative stress and inflammatory response (17). Furthermore, carnosic acid also exerts important roles in neurodegenerative diseases by regulating oxidative stress and microglia-mediated neuroinflammation (18). Notably, recent study confirms the protective efficacy of carnosic acid in rheumatoid arthritis by suppressing inflammation, astrogliogenesis, bone resorption and joint injury (19, 20). Furthermore, carnosic acid can inhibit macrophage activation-evoked inflammatory response (21). Up to now, the roles of carnosic acid in OA remain unclear. Based on the pathological characteristics of OA (oxidative stress and inflammatory response) and the potential anti-inflammation and anti-oxidative function of carnosic acid, we hypothesize that carnosic acid may attenuate the progression of OA by regulating oxidative stress and inflammation evoked by macrophages. In the present research, we sought to elaborate the function of carnosic acid in macrophage polarization-mediated inflammation and oxidative injury. Moreover, we also investigated its effects on OA model to further corroborate our hypothesis.

## 2 Materials and methods

### 2.1 Macrophage culture and treatments

Mouse macrophage line Raw 264.7 was purchased from the Procell Life Science & Technology (Wuhan, China). For culture, Raw 264.7 cells were incubated with RPIM 1640 medium containing 10% (v/v) fetal bovine serum (FBS), and 1% penicillin/streptomycin. Cells (5×10^5^ cells/ml) were treated with various doses of carnosic acid (1-100 μM; Sigma, St. Louis, MO, USA) dissolved in 0.1% dimethyl sulfoxide (DMSO) in saline. To induce macrophage polarization towards M1-like phenotype, Raw264.7 cells were exposed to 100 ng/ml LPS (Sigma) for 24 h as previously reported (11, 22). For macrophage M2-like polarization, cells were cultured with medium supplemented with 20 ng/mL IL-4 (Sigma) for 24 h as previously reported (11, 22). Then, to prepare the conditioned medium (CM) from macrophages, the original medium was replaced with a fresh serum-free medium for 24 h. After centrifugation with a speed of 1000 × g for 5 min, the supernatants were collected and served as CM for chondrocyte culture. All cells were incubated under 95% air and 5% CO_2_ condition at 37°C.

### 2.2 Immunofluorescence assay

Raw 264.7 cells were stimulated with the indicated conditions. For immunofluorescence analysis, cells were fixed with 4% paraformaldehyde for 0.5 h, followed by permeabilization with 0.3% Triton X-100 for 15 min. After rinsing with PBS three times, cells were cultured in medium containing 1% bovine serum albumin. One hour later, cells were incubated with primary antibody against M1 macrophage marker CD86 and M2-like macrophage marker CD206 (Abcam, Cambridge, MA, USA) overnight at 4°C. Subsequently, cells were treated with fluorescein isothiocyanate-conjugated second antibody for 1 h. Then, cells were incubated with DAPI for 10 min under dark to stain cellular nuclear. Finally, all specimens were visualized using an Olympus fluorescence microscope. The key antibody information was shown in **Supplementary Table S1**.

### 2.3 Knockdown of Nrf2

For inhibition of Nrf2 expression, macrophages in a 24-well plate at a density of 5×10^5^ cells/well were transfected with 100 nM si-Nrf2 or scrambled siRNA using Lipofectamine RNAiMAX reagent (Invitrogen, Camarillo, CA, USA). The scrambled siRNAs were defined as the negative control (si-NC). The siRNA sequences targeting Nrf2 (cat. sc-37049) and si-NC (cat. sc- 37007) were synthesized from Santa Cruz Biotechnology, Inc. (Santa Cruz, CA, USA). Forty-eight hours later, the expression of Nrf2 protein was analyzed by western blotting.

### 2.4 Experimental animals and ethics statement

Thirty-six male C57BL/6 mice (8–10 weeks old) were bought from the Center of Laboratory Animals, the Fourth Military Medical University. In this study, all animal protocols complied with the National Institutes of Health (NIH) Guide for the Care and Use of Laboratory Animals, and approved by the Animal Care and Use Committee of Zhongda Hospital, School of medicine, Southeast University Hospital (No. 2023ZDSYLL132-A01). Before the experiments, all mice were acclimated for 7 days and housed in a standard condition (a 12 h light–dark cycle, constant temperature of 23 ± 2°C, and 55-60% humidity) with ad libitum access to food and water. During the animal experiments, all reasonable efforts were performed to minimize suffering.

### 2.5 Chondrocytes isolation and culture

Mice primary chondrocytes were isolated from mice articular cartilage as previously described (11). Articular cartilage was collected from mice and cut into 1 mm^3^ pieces. After digestion with 0.25% trypsin for 0.5 h, specimens were then treated with 0.2% collagenase II at 37°C for 5 h. Then, cells were filtered using a Corning of 40 μm cell filter (Sigma) and rinsed with PBS. Afterward, the collected chondrocytes were maintained in DMEM medium containing 10% FBS under 5% CO_2_ at 37°C. The isolated chondrocytes were passed at 80% confluence. The prepared chondrocytes were identified by immunofluorescence staining with collagen II (Santa Cruz Biotechnology; Santa Cruz, CA, USA) (11) and more than 95% of cells were judged to be chondrocytes. The cells from passage 1 to 2 were used for the subsequent experiments. For co-culture, the CM from M1-like macrophages was diluted at the ratio of 1:1 with serum-free medium and added to chondrocytes for further analyses for 48 h.

### 2.6 Cell viability assay

Macrophages (5×10^5^ cells/well) were treated with various concentrations of carnosic acid for 24 h. Chondrocytes, at a concentration of 5×10^3^ cells/well in 96-well plates, were stimulated with CM from macrophages under LPS and carnosic acid conditions. Then, cells were collected and incubated with 10 μl of a Cell Counting Kit (CCK)-8 solution (Nanjing Jiancheng Bioengineering Institute, Nanjing, China). Three hours later, all samples were analyzed using a spectrophotometer to measure an absorbance at 450 nm to evaluate cell viability.

### 2.7 qRT-PCR

Total RNA from cells was isolated by a TRIzol reagent, followed by a synthesis of first cDNA using the Prime Script-RT Reagent Kit (TaKaRa, Dalian, China). Then, cDNA was used as a template to perform the real-time PCR amplification to determine mRNA levels of CD86, IL-1β, TNF-α, IL-6, iNOS, CD206, CD163, IL-10, aggrecan, collagen II, ADAMTS5, MMP-3, and MMP-13. All experimental protocols were conducted in accordance with instructions of a commercial SYBR Premix Ex TaqTM II Kit (TaKaRa). The specific primers for above genes were synthesized by the Sangon Biotech (Shanghai, China) and shown in **Supplementary Table S2**. All transcriptional levels of genes were analyzed with β-actin for normalization and 2^−ΔΔCT^ method.

### 2.8 Administration of OA mice model

Anterior cruciate ligament transection (ACLT) surgery was performed to construct the OA model as previously reported (11). Mice were randomly divided into the following groups (n=6 in each group) using the random number table method: sham groups, OA groups, OA and carnosic acid groups. Furthermore, the investigators were blinded to group allocation and evaluation of outcome after different groups were administrated. In the OA groups, mice underwent ACLT surgery in the right knee. Mice in sham groups were performed by only opening joint capsule and suturing the incision but without ACLT surgery. In the OA and carnosic acid groups, OA mice were administrated intra-articularly with carnosic acid (dissolved in saline with 0.1% DMSO solution) starting from the first day after surgery at doses of 5 mg/kg, 10 mg/kg and 20 mg/kg, 50 mg/kg (10 μL) twice a week for 8 weeks. During this process, the other groups received an equal volume of DMSO in saline.

### 2.9 Histological evaluation

Mice were anesthetized intraperitoneally with pentobarbital sodium at a dose of 50 mg/kg and euthanized by cervical dislocation to collect articular cartilage and synovial tissues. Then, the prepared cartilage of knee joint was fixed, dehydrated in a graded ethanol series and embedded in paraffin. Subsequently, samples were cut into serial 5-μm thick sections. Then, sections were stained with H&E and safranin O-fast green. Cartilage degeneration was evaluated by an Osteoarthritis Research Society International (OARSI) scoring system. Joint synovitis was assessed by the synovitis scoring system (11, 22). Two pathologists with over 5 years of experience in animal pathology blinded to group allocation and were involved in evaluating pathological changes from at least five randomly selected fields of articular cartilage samples.

### 2.10 Western assay

Samples from cells and articular cartilage tissues were lysed with the appropriate RIPA lysis buffer (Beyotime, Shanghai, China) to extract total protein. Proteins in the nucleus were prepared according to the instructions of a Nuclear Protein Extraction Kit (Beyotime, Shanghai, China). Then, equal amounts of protein (25 μg) were loaded onto 10% SDS-PAGE and subsequently transferred onto PVDF membranes. The membranes were then incubated with primary antibodies against mouse collagen II (Santa Cruz Biotechnology; Santa Cruz, CA, USA), aggrecan, ADAMTS5, Nrf2, HO-1, p-p65 NF-κB, p65 NF-κB, Bax, and Bcl-2 (all from Abcam, Cambridge, MA, USA) at 4°C. Following incubation with secondary antibodies conjugated to horseradish peroxidase, the binding proteins were analyzed by a ECL reagent (Beyotime) and Image J software. β‐actin or Lamin B was used as the internal control. The key antibody information was shown in **Supplementary Table S1**.

### 2.11 Measurement of inflammatory cytokine and MMP levels

Inflammatory cytokine (IL-1β, IL-6, TNF-α, and IL-10) were quantified using the commercial ELISA Kits (Beyotime). The concentration of NO in chondrocytes was measured using an NO detection kit (Nanjing Jiancheng Bioengineering Institute, Nanjing, China). The levels of inflammatory mediators PGE2, MMP-3, and MMP-13 in chondrocytes and tissues were assessed using the corresponding commercial ELISA kits (eBioscience, San Diego, CA, USA). All procedures were performed in accordance with the manufacturer’s protocols.

### 2.12 Macrophage polarization assay

Synovial tissues of mice were collected and digested with Hank’s balanced Salt solution containing collagenase II. Approximately 0.5 h later, samples were filtered and centrifuged for 5 min, and re-suspended in 500 µl of icy Hank’s balanced Salt Solution. Then, specimens were treated with fluorescently labeled anti-mouse F4/80, CD86 and CD206 (BioLegend, San Diego, CA, USA) on ice. Thirty minutes later, all samples were analyzed using a FACSCalibur flow cytometer. Samples were first gated by forward light scatter (FSC) versus side scatter (SSC) and pulse-width to exclude debris and aggregates. Macrophages were sorted based on F4/80^+^ gating. Then, CD86^+^F4/80^+^ M1 macrophages and CD206^+^F4/80^+^ M2 macrophages were sorted.

### 2.13 Measurements of ROS, malondialdehyde and superoxide dismutase

For the detection of ROS in chondrocytes, cells (5×10^3^ cells) were incubated with cell-permeative probe 2’, 7’-dichlorodihydrofluorescein diacetate (DCFH-DA; 10 µM, Beyotime) for 30 min to yield fluorescent DCF. Then, samples were analyzed using a fluorometric microplate reader.

To measure SOD and MDA levels, the supernatants from cells and tissues were quantified using the commercial SOD and MDA Detection Kits (Nanjing Jiancheng Bioengineering Institute). All procedures were carried out according to the manufacturer’s instructions.

### 2.14 Statistical analysis

In this study, power calculations were carried out to define group sizes with a statistical power of 80% and α level set at 0.05, leading to a sample size of n = 6 in each group. All experiments were carried out in triplicate and repeated at least three times. All data were obtained from at least three-independent experiments and analyzed by GraphPad Prism software. Shapiro-Wilk and Levene tests were applied to analyze the normal distribution and homogeneity of data, respectively. Non-parametric data (such as the OARSI scores) were analyzed using a Kruskal-Wallis test. For variables that exhibited a normal distribution, the results are presented as the mean ± standard deviation (SD). The statistical significance for multiple groups was analyzed by ANOVA with post-Tukey’s test and Student t-test was applied for analysis between two groups. *P* < 0.05 were denoted as statistically significant.

## 3 Results

### 3.1 Carnosic acid treatment affects macrophage polarization from M1 phenotype to M2-like phenotype

**Figure 1:**
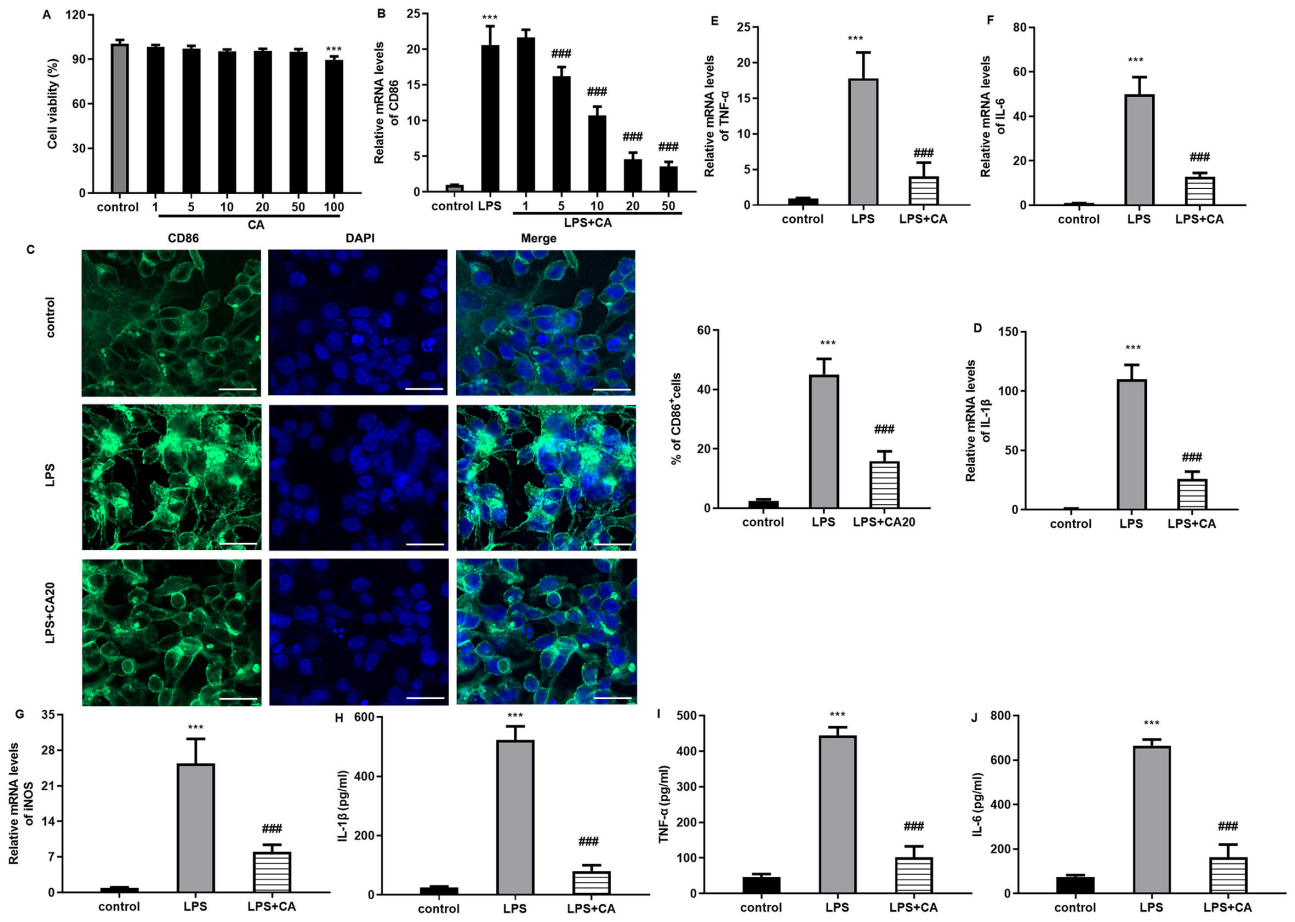
Carnosic acid inhibits macrophage M1 polarization. **(A)** Raw264.7 cells were treated with the indicated doses of carnosic acid. Then, cell viability was determined. **(B)** Cells were stimulated with various doses of carnosic acid under LPS exposure. Then, the mRNA levels of M1 macrophage marker CD86 were detected. **(C)** The percentage of CD86^+^ macrophages was analyzed by immunofluorescence assay. Scale bar=100 μm. **(D–G)** The transcription levels of IL-1β **(D)**, TNF-α **(E)**, IL-6 **(F)**, and iNOS **(G)** were determined. **(H–J)** The levels of M1 macrophage-related inflammatory cytokine IL-1β **(H)**, TNF-α **(I)**, IL-6 **(J)** were analyzed. ^***^P < 0.001 vs. control group, ^###^*P* < 0.001 vs. LPS groups.

Before investigating the effects of carnosic acid on macrophage polarization, we first analyzed its toxicity on macrophages. As shown in **Figure 1A**, carnosic acid showed little toxicity to Raw264.7 cell viability at doses ranging from 1 to 50 μM, but obviously inhibited cell viability at 100 μM. After exposure to LPS, the transcription levels of the M1-like macrophage marker CD86 were elevated, which were dose-dependently inhibited by carnosic acid; moreover, no obvious difference was observed between the 20 μM and 50 μM carnosic acid groups (**Figure 1B**). Thus, 20 μM of carnosic acid was chosen for subsequent experiments. Further immunofluorescence assay revealed that compared with the control groups, the increased amounts of CD86^+^ M1 macrophages were validated in LPS-treated groups, which were reversed by carnosic acid (**Figure 1C**). Concomitantly, the LPS stimulation enhanced mRNA levels of M1 macrophage markers relative to the control groups, including IL-1β (**Figure 1D**), TNF-α (**Figure 1E**), IL-6 (**Figure 1F**), and iNOS (**Figure 1G**). Furthermore, LPS also elevated the contents of M1 macrophage-related inflammatory cytokine IL-1β, TNF-α and IL-6 in supernatants (**Figures 1H–J**). However, these increases were attenuated after carnosic acid treatment, indicating that carnosic acid may suppress LPS-driven macrophage polarization towards M1-like phenotype.

**Figure 2:**
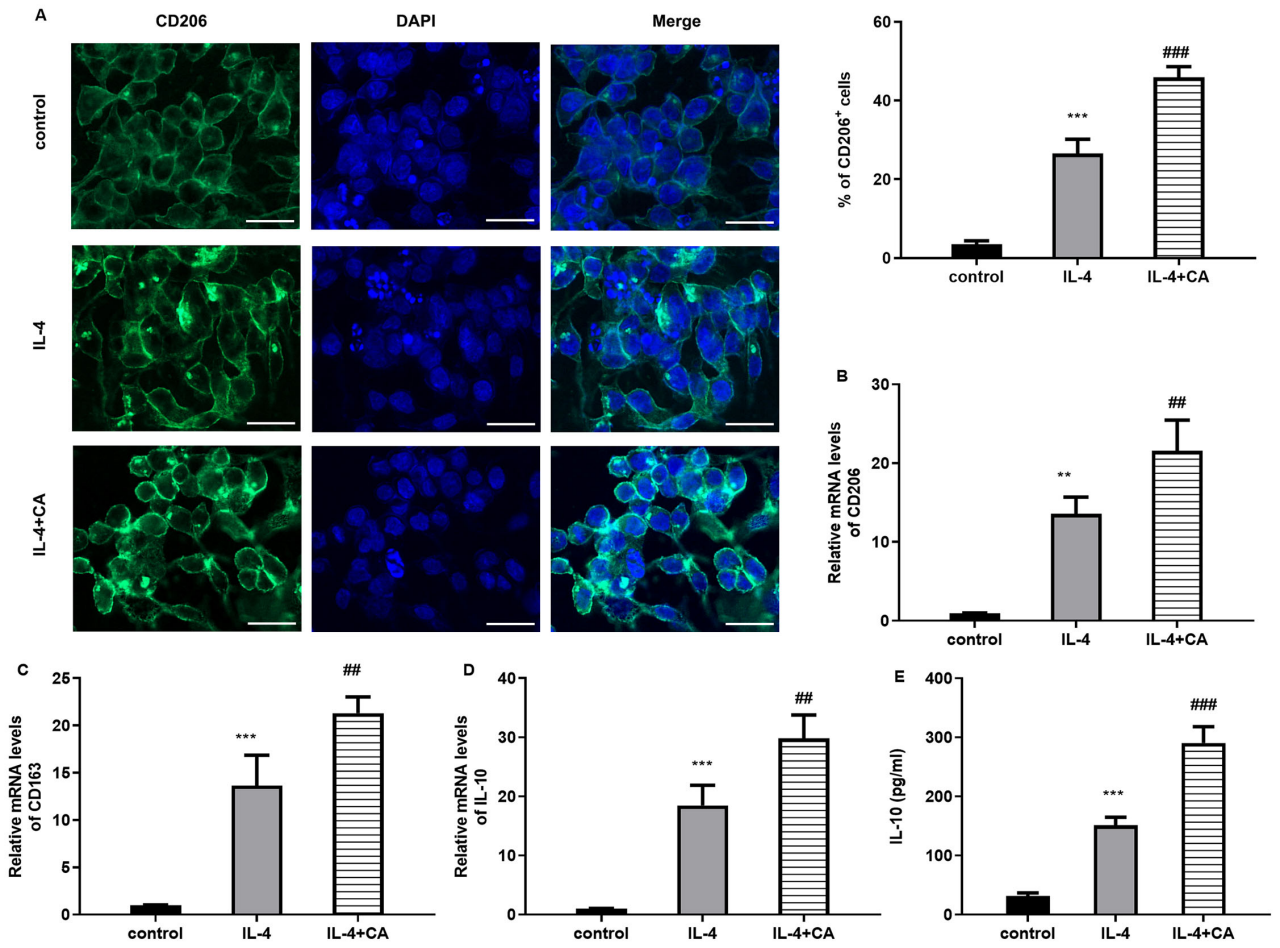
Treatment with carnosic acid increases macrophage M2-like polarization. Raw264.7 macrophages were treated with carnosic acid under IL-4 stimulation. Then, the % of CD206^+^ M2-like macrophages was evaluated by immunofluorescence analysis. Scale bar=100 μm. **(B–D)** QRT-PCR assay was performed to measure the mRNA levels of CD206 **(B)**, CD163 **(C)**, and IL-10 **(D)**. **(E)** The contents of IL-10 were quantified by ELISA assay. ^**^P < 0.01, ^***^P < 0.001 vs. control group, ^##^*P* < 0.01, ^###^*P* < 0.001 vs. IL-4 groups.

Compared with the control groups, IL-4 stimulation enhanced the amounts of CD206^+^ M2-like macrophages, which were further increased by carnosic acid (**Figure 2A**). Moreover, IL-4 increased the mRNA expression of M2 macrophage marker CD206 (**Figure 2B**), CD163 (**Figure 2C**) relative to the control groups, as well as expression of anti-inflammatory IL-10 (**Figures 2D, E**). Notably, carnosic acid further increased IL-4-induced expression of M2-like macrophage markers (**Figures 2B–E**). Thus, carnosic acid may enhanced macrophage M2-like polarization.

### 3.2 Carnosic acid alleviates macrophage conditional medium-evoked chondrocyte oxidative injury and inflammation

**Figure 3:**
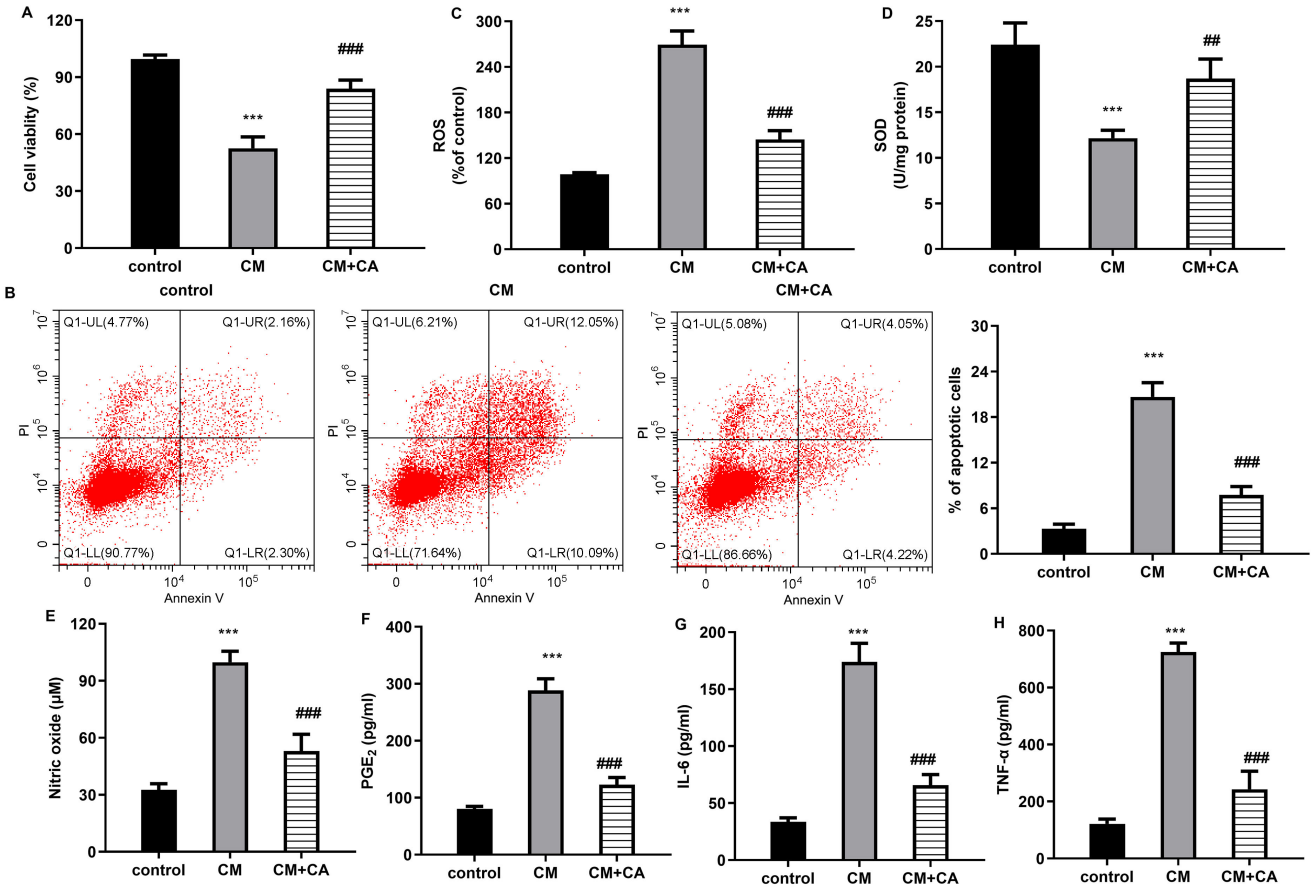
Carnosic acid attenuates macrophage conditional medium (CM)-evoked chondrocyte oxidative injury and inflammation. **(A)** Chondrocytes were cultured with CM from LPS-induced M1macrophage (CM group) under carnosic acid condition (CM+carnosic acid group). Then, cell viability was analyzed. **(B)** Annexin V/PI staining was performed to detect cell apoptosis. **(C–H)** The levels of ROS **(C)**, SOD **(D)**, NO **(E)**, PGE2 **(F)**, IL-6 **(G)** and TNF-α **(H)** were further determined. ^***^P < 0.001 vs. control group, ^##^*P* < 0.01, ^###^*P* < 0.001 vs. CM groups.

Accumulating studies reveal the key roles of macrophage polarization in the progression of OA. Thus, we next explored the efficacy of carnosic acid in macrophage-mediated chondrocyte dysfunction. As shown in **Figures 3A, B**, treatment with CM from LPS-induced macrophages obviously suppressed viability (**Figure 3A**) and enhanced apoptosis of chondrocytes (**Figure 3B**), which were offset by carnosic acid. Concomitantly, the increased ROS levels (**Figure 3C**) and decreased anti-oxidative SOD levels (**Figure 3D**) were observed in chondrocytes cultured with CM from LPS-induced M1 macrophages; nevertheless, these changes were overturn when cells were incubated with CM from carnosic acid-treated macrophages under LPS conditions. Furthermore, the increased levels of inflammatory mediator NO (**Figure 3E**), PGE2 (**Figure 3F**) and cytokine IL-6 (**Figure 3G**) and TNF-α (**Figure 3H**) in chondrocytes incubated with CM from LPS-induced macrophages were inhibited by carnosic acid. Thus, these data suggest that carnosic acid may protect against M1 macrophages-mediated oxidative injury and inflammation in chondrocytes.

### 3.3 Carnosic acid antagonizes macrophage conditional medium-evoked ECM degradation in chondrocytes

**Figure 4:**
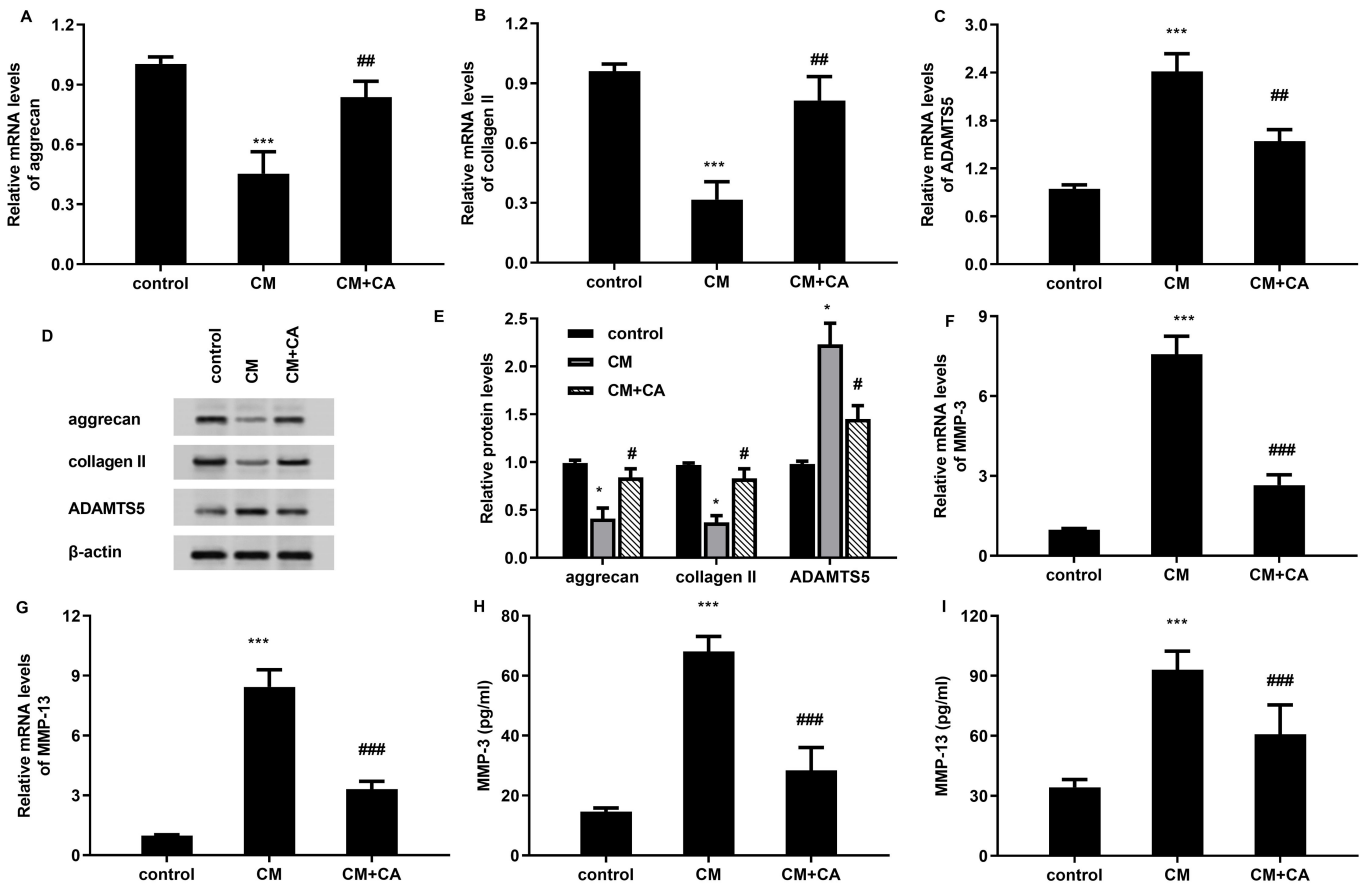
Carnosic acid abrogates macrophage-evoked ECM degradation in chondrocytes. **(A-C)** After incubation with CM from LPS-treated macrophages under carnosic acid conditions, the mRNA levels of aggrecan **(A)**, collagen II **(B)**, and ADAMTS5 **(C)** were analyzed by qRT-PCR. **(D, E)** The corresponding protein levels of aggrecan, collagen II, and ADAMTS5 were determined by western blotting assay and Image J software. **(F, G)** The mRNA levels of MMP-3 **(F)** and MMP-13 **(G)** were detected. **(H, I)** The production of MMP-3 and MMP-13 was quantified. ^***^P < 0.001 vs. control group, ^##^*P* < 0.01, ^###^*P* < 0.001 vs. CM groups.

Further assay substantiated that co-culture with LPS-treated macrophages dramatically inhibited mRNA levels of cartilage ECM anabolic component aggrecan (**Figure 4A**), collagen II (**Figure 4B**), and increased ADAMTS5 transcript (**Figure 4C**). Concomitantly, the increased transcriptional and protein expression of aggrecan, collagen II, ADAMTS5 (**Figures 4A–E**) in CM-treated chondrocytes were suppressed when cells were incubated with CM from carnosic acid-treated M1 macrophages. Additionally, macrophage CM also enhanced the mRNA expression (**Figure 4F, G**) and production (**Figures 4H, I**) of ECM catabolism-related MMP-3 and MMP-13 in chondrocytes. Thus, carnosic acid may affect M1 macrophage-evoked metabolism disorder of chondrocytes.

### 3.4 Treatment with carnosic acid restrains the activation of the Nrf2/NF-kB axis

**Figure 5:**
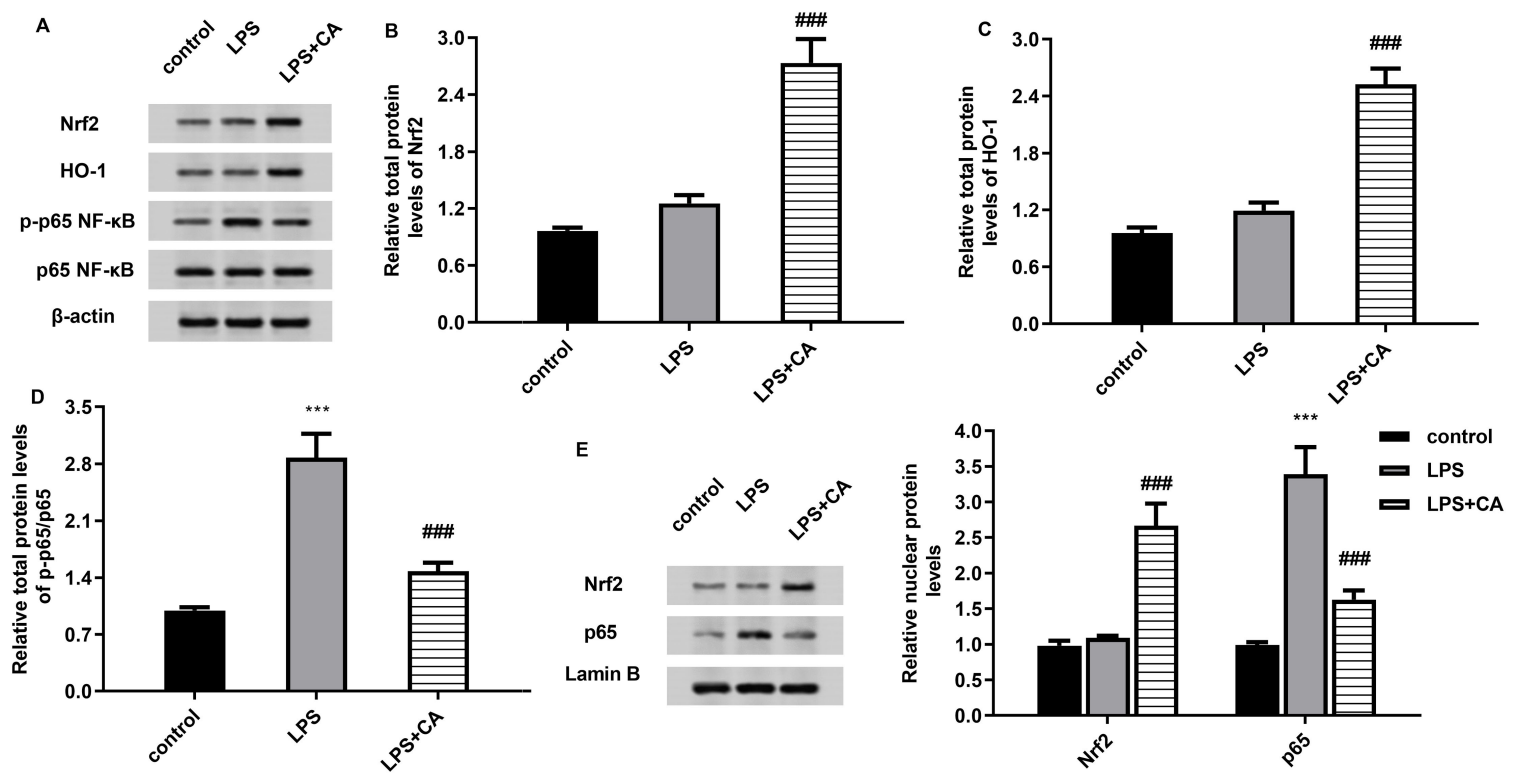
Carnosic acid inhibits activation of the Nrf2/NF-kB axis. **(A–D)** Following stimulation with LPS and carnosic acid, the protein levels of Nrf2, HO-1, p-p65 NF-kB and p65 NF-kB in macrophages were analyzed by western. The corresponding bands were quantified by Image J software. **(E)** The protein levels of nuclear Nrf2 and p65 in cells under LPS and carnosic acid conditions. ^***^P < 0.001 vs. control group, ^###^*P* < 0.001 vs. LPS groups.

Abundant evidence confirms the critical roles of Nrf2/NF-kB axis in the development of OA. In this study, carnosic acid treatment further increased the protein expression of total Nrf2, HO-1 in LPS-treated macrophages (**Figures 5A, C**). Moreover, the increased expression of downstream p-p65 NF-κb in LPS-treated cells were suppressed by carnosic acid (**Figure 5A, D**). Moreover, carnosic acid increased the nuclear protein expression levels of Nrf2 and inhibited nuclear p65 expression in cells under LPS condition (**Figure 5E**). Therefore, these findings indicate that carnosic acid may inhibit LPS-activated p65 NF-κb signaling by activating the Nrf2 pathway.

### 3.5 Reactivation of the Nrf2/NF-kB axis reverses carnosic acid-mediated chondroprotective efficacy from macrophage M1 polarization condition

**Figure 6:**
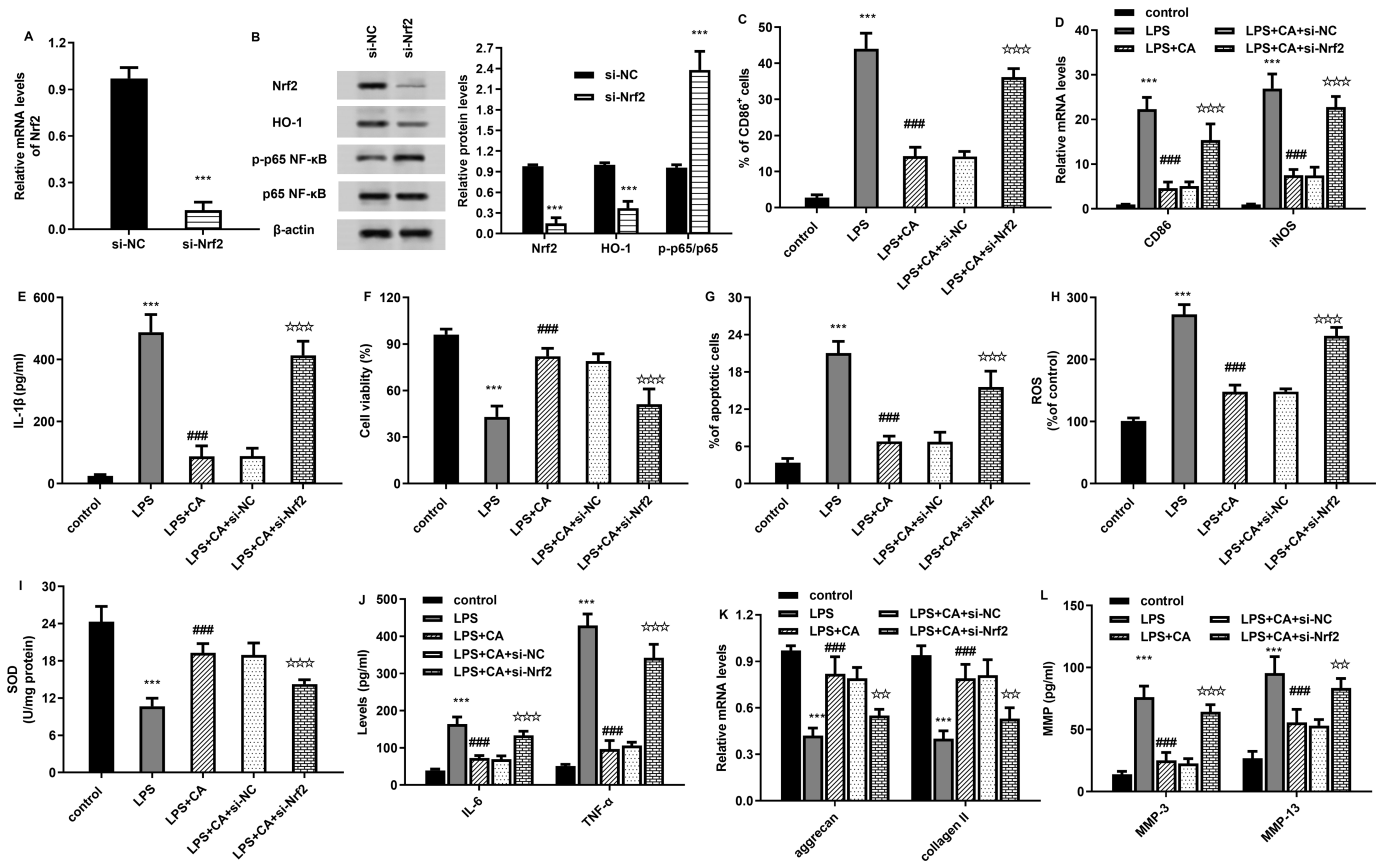
The Nrf2/NF-kB axis is responsible for carnosic acid-mediated chondroprotective efficacy from macrophage M1 polarization condition. **(A)** After transfection with si-Nrf2, the mRNA levels of Nrf2 were determined by qRT-PCR. **(B)** The effects of si-Nrf2 transfection on the activation of the Nrf2/NF-kB axis in macrophages. **(C–E)** Macrophages were transfected with si-Nrf2 under LPS and carnosic acid conditions. Then, the % of CD86^+^ M1 macrophages **(C)**, mRNA levels of CD86, iNOS, **(D)** and IL-1β **(E)** were assessed. **(F–L)** After incubation with CM from macrophages under si-Nrf2, LPS and carnosic acid conditions, the viability **(F)** and apoptosis **(G)** of chondrocytes were detected. Then, the levels of ROS **(H)**, SOD **(I)**, IL-6 and TNF-α **(J)**, mRNA expression of aggrecan, collagen II **(K)**, levels of MMPs **(L)** were further determined. ^***^P < 0.001 vs. control group, ^###^*P* < 0.001 vs. LPS groups, ^☆☆^*P* < 0.01, ^☆☆☆^*P* < 0.001 vs. LPS and CA groups.

After transfection with si-Nrf2, the mRNA levels of Nrf2 were significantly decreased to 0.12-fold (**Figure 6A**). Moreover, si-Nrf2 transfection also obviously inhibited protein expression of Nrf2, HO-1 and increased the p-p65 NF-kB expression (**Figure 6B**). Moreover, LPS-increased percentages of CD86^+^ macrophages were reduced after carnosic acid treatment, which were reversed after Nrf2 down-regulation (**Figure 6C**). Concomitantly, targeting Nrf2 pathway also offset the inhibitory effects of carnosic acid on LPS-elevated M1 macrophage marker expression of CD86, iNOS (**Figure 6D**) and IL-1β (**Figure 6E**). Furthermore, carnosic acid attenuated LPS-induced oxidative injury of chondrocytes by increasing cell viability (**Figure 6F**), inhibiting cell apoptosis (**Figure 6G**), ROS levels (**Figure 6H**) and enhancing anti-oxidative SOD levels (**Figure 6I**), which were overturn by down-regulation of NRf2. Additionally, blockage of the Nrf2 signaling also reversed carnosic acid-inhibited inflammatory cytokine levels of IL-6 and TNF-α (**Figure 6J**), and aggrecan, collagen II expression (**Figure 6K**) in chondrocytes under LPS-induced macrophage condition. Moreover, targeting Nrf2 pathway also inversed the effects of carnosic acid on the levels of MMP-3 and MMP-13 in chondrocytes co-cultured with CM from LPS-treated macrophages (**Figure 6L**).

### 3.6 Treatment with carnosic acid alleviates the progression of OA in mice model

**Figure 7:**
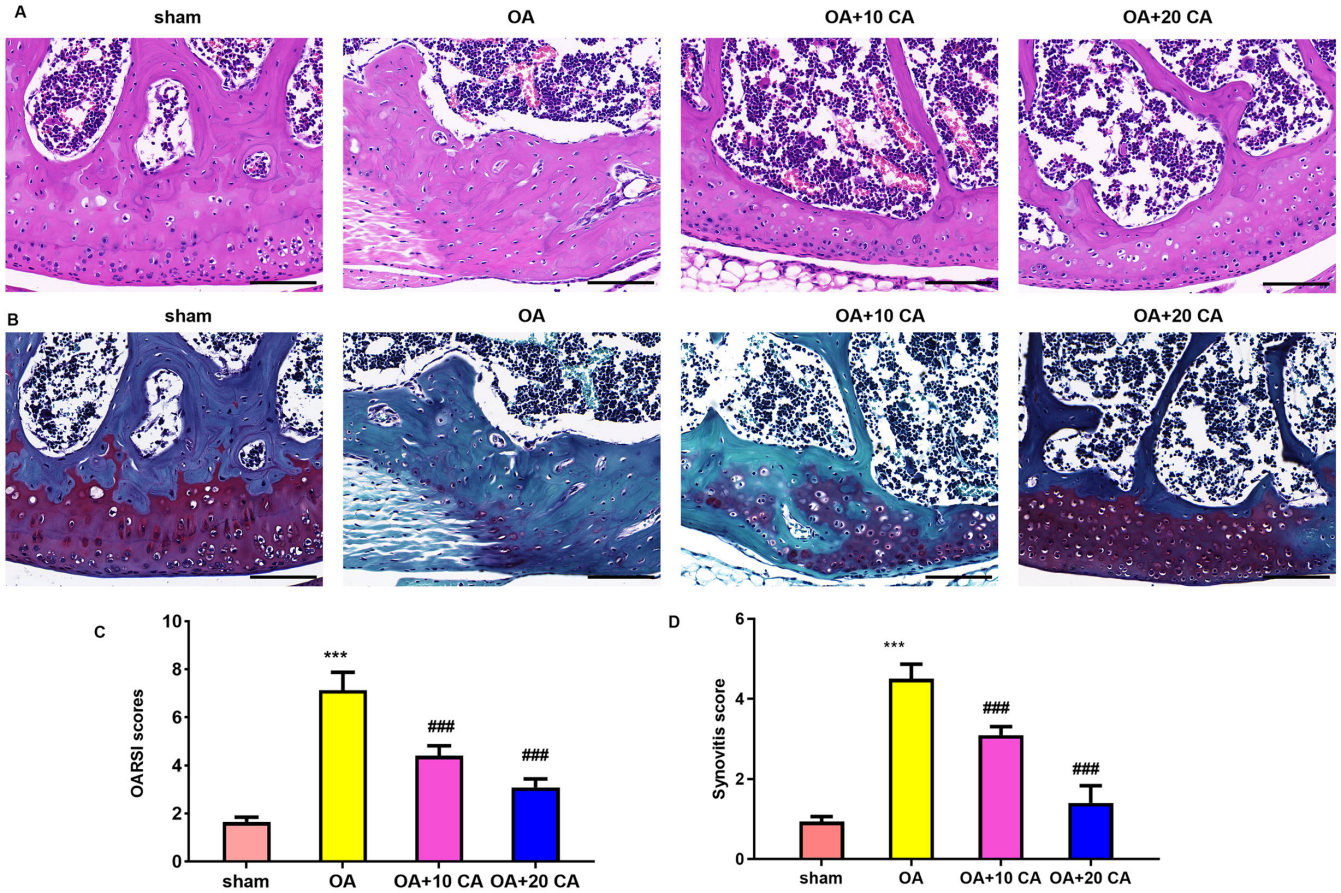
Administration with carnosic acid attenuates the progression of OA model. OA model was administrated with carnosic acid for 8 weeks. Then, HE **(A)** and Safranin O staining **(B)** were performed. Scale bar=100 μm. Furthermore, the OARSI scores **(C)** and synovitis scores **(D)** were also analyzed. ^***^P < 0.001 vs. sham group, ^###^*P* < 0.001 vs. OA groups.

We next explored the function of carnosic acid in OA model *in vivo*. Notably, the preliminary data confirmed that the increased OARSI scores in OA groups were significantly reduced after treatment with 10, 20 and 50 mg/kg of carnosic acid, indicating the efficacy of carnosic acid in alleviating the severity of OA; however, no obvious changes were found between the 20 and 50 mg/kg of carnosic acid groups (**Supplementary Figure S1**). Therefore, 10 and 20 mg/kg of carnosic acid was chosen for the following experiments. As shown in **Figures 7A, B**, HE (**Figure 7A**) and Safranin O staining (**Figure 7B**) corroborated the obvious reduction of cartilage matrix and thickness in OA mice model relative to the sham groups, which were attenuated by carnosic acid. Furthermore, administration with carnosic acid also dose-dependently reduced OARSI scores (**Figure 7C**) and synovitis scores (**Figure 7D**) in OA model, indicating the attenuation of cartilage degeneration and severe synovitis in OA. Thus, these data reveal that carnosic acid can attenuate the progression of OA.

### 3.7 Administration with carnosic acid attenuates oxidative injury and cartilage degradation in mice with OA

**Figure 8:**
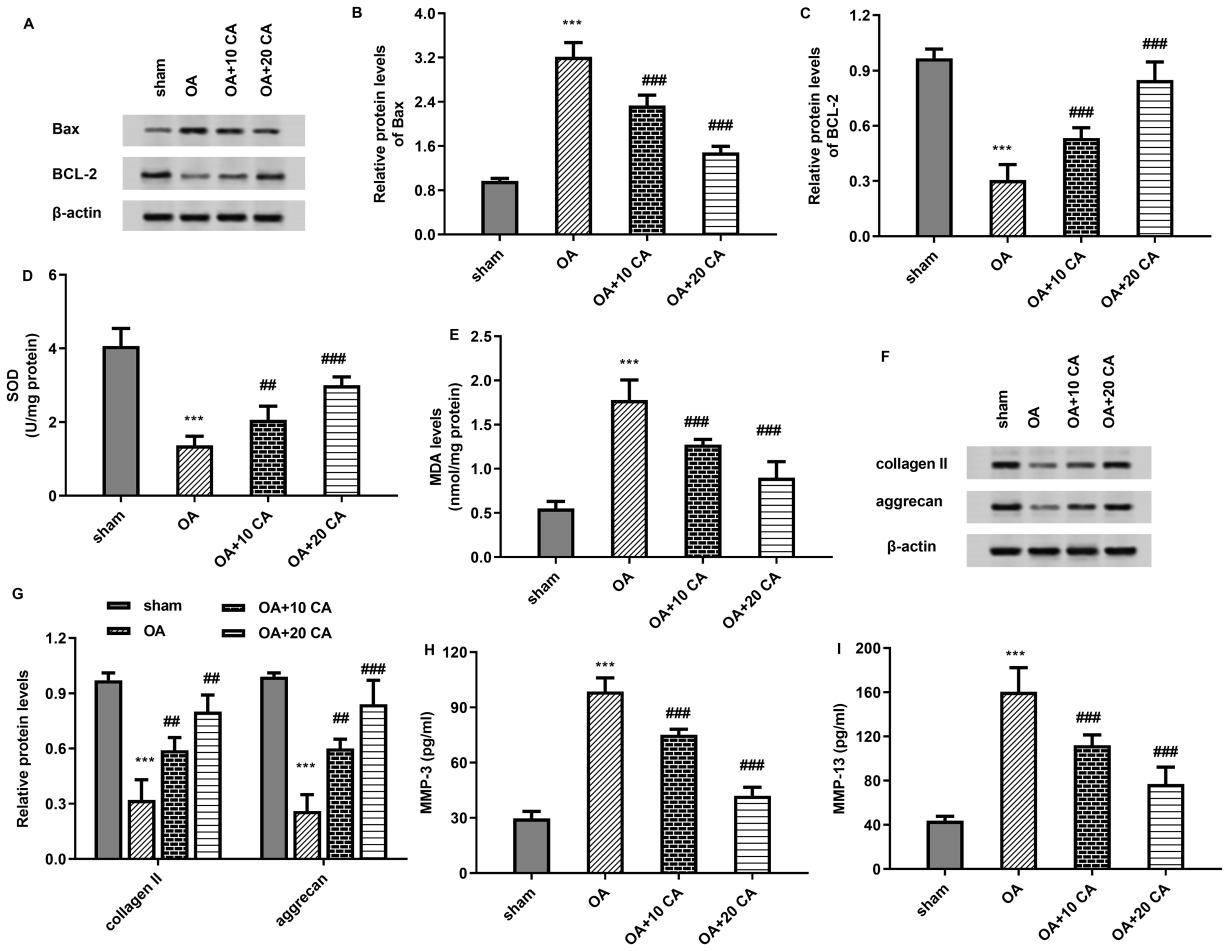
Carnosic acid alleviates oxidative injury and cartilage degradation in OA model. **(A–C)** OA mice were administrated with the indicated doses of carnosic acid. Then, the protein expression of Bax and Bcl-2 was analyzed. **(D, E)** The efficacy of carnosic acid on oxidative stress-related SOD **(D)** and MDA **(E)** were determined in OA model. **(F, G)** Western assay was carried out to determine the protein levels of collagen II and aggrecan. **(H, I)** The contents of MMP-3 **(H)** and MMP-13 **(I)** were detected by commercial kits. ^***^P < 0.001 vs. sham group. ^##^*P* < 0.01, ^###^*P* < 0.001 vs. OA groups.

As shown in **Figures 8A–C**, the increased protein expression of pro-apoptotic Bax and decreased expression of anti-apoptotic Bcl-2 were validated in articular cartilage tissues from OA mice, which were dose-dependently attenuated by carnosic acid. Furthermore, OA mice showed decreased levels of SOD (**Figure 8D**) and increased levels of MDA (**Figure 8E**); however, these changes were offset after carnosic acid administration. Additionally, compared with the sham groups, the protein expression of collagen II and aggrecan was suppressed in OA groups, which were reversed by carnosic acid (**Figures 8F, G**). Moreover, the up-regulated levels of MMP-3 (**Figure 8H**) and MMP-13 (**Figure 8I**) in OA mice were inhibited following treatment with carnosic acid.

### 3.8 Treatment with carnosic acid inhibits macrophage M1 polarization, inflammation and Nrf2/NF-kB axis signaling *in vivo*

**Figure 9:**
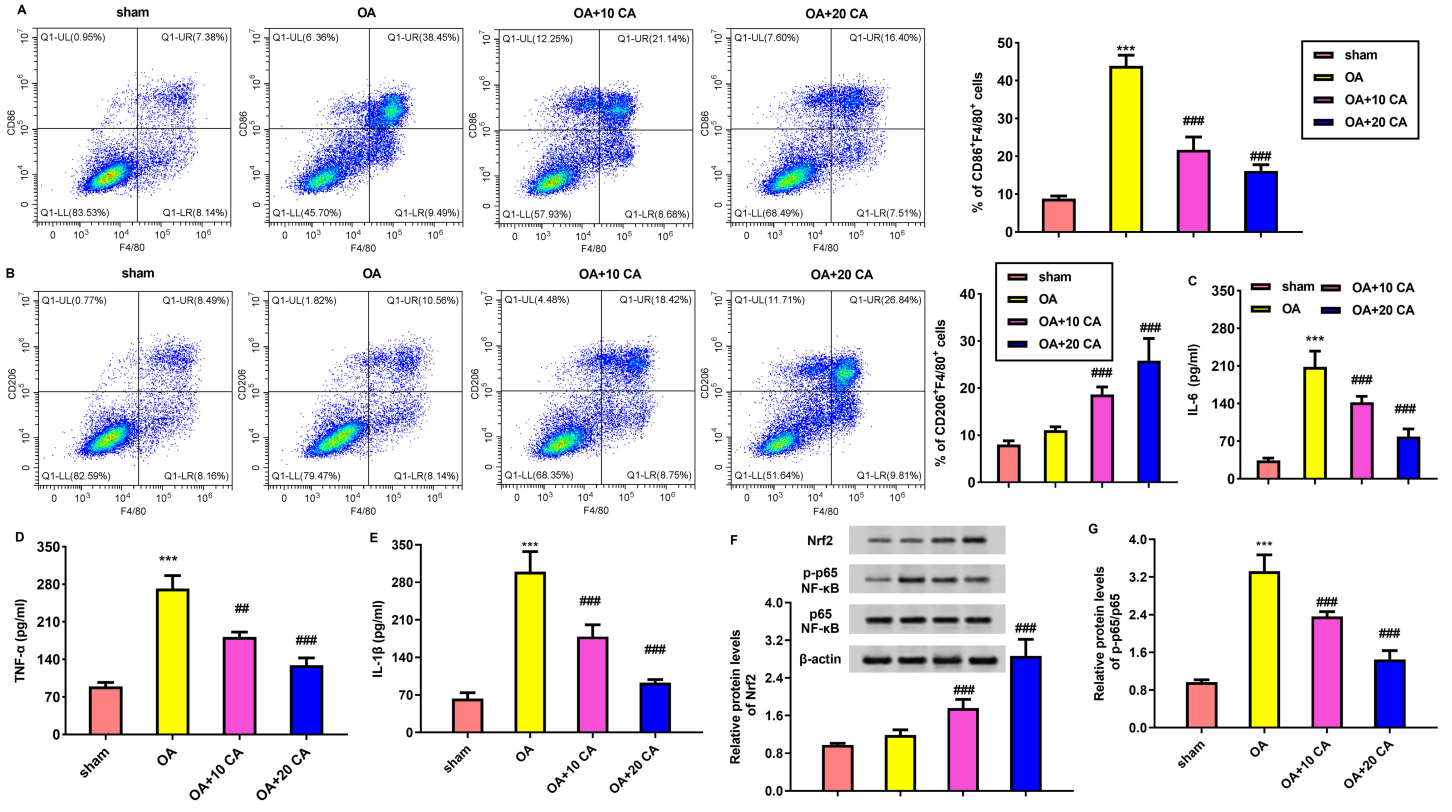
Carnosic acid suppresses macrophage M1-like polarization, inflammation and Nrf2/NF-kB axis in OA mice. **(A, B)** OA mice were administrated with the indicated concentration with carnosic acid. Eight weeks later, the % of CD86^+^ F4/80^+^ M1 macrophages **(A)** and CD206^+^ F4/80^+^ M2 macrophages in synovium were analyzed by flow cytometry. **(C–E)** The effects on cytokine levels of IL-6 **(C)**, TNF-α **(D)**, IL-1β **(E)** were analyzed. **(F, G)** The activation of Nrf2/NF-kB axis was detected in articular cartilage tissues. ^***^P < 0.001 vs. sham group. ^##^*P* < 0.01, ^###^*P* < 0.001 vs. OA groups.

Further assay confirmed the increased percentages of CD86^+^ F4/80^+^ M1 macrophages in OA mice relative to the sham groups (**Figure 9A**). Notably, administration with carnosic acid dose-dependently reduced percentages of CD86^+^ F4/80^+^ M1 macrophages and up-regulated percentages of CD206^+^ F4/80^+^ M2 macrophages in synovium (**Figure 9B**), in contrast to the sham groups. Additionally, compared with the sham groups, higher pro-inflammatory cytokine levels of IL-6 (**Figure 9C**), TNF-α (**Figure 9D**), IL-1β (**Figure 9E**) were observed in OA mice, which were abrogated by carnosic acid. Mechanically, treatment with carnosic acid enhanced the protein expression of Nrf2 and suppressed protein levels of p-p65 NF-κb in OA mice (**Figure 9F, G**).

## 4 Discussion

Osteoarthritis (OA) is a chronic inflammatory and degenerative joint disease that predominantly affects middle-aged and elderly population. Carnosic acid, a natural active component, has been widely accepted to exert the anti-inflammatory properties in several inflammation-related diseases (16, 17). However, its roles in OA remain unclear. In this study, carnosic acid reprogramed macrophage polarization by shifting the pro-inflammatory M1-like phenotype toward anti-inflammatory M2-like phenotype. Moreover, carnosic acid protected chondrocytes against oxidative injury, inflammation and ECM metabolic disorders induced by M1 macrophage-conditioned medium. Importantly, carnosic acid alleviated articular cartilage degeneration and synovitis macrophage-evoked inflammation and cartilage damage in ACLT-mimicked mouse model of OA. Therefore, these findings may support carnosic acid as a promising therapeutic agent for OA.

Articular inflammation is majorly characterized by synovitis that is closely associated with the initiation and progression of OA (7, 23). Macrophages are crucial immune cells within the synovium and exert critical roles in synovial inflammation by their polarization (23). Intriguingly, accumulating evidence has revealed the increased levels of pro-inflammatory M1-polarized macrophages and decreased levels of anti-inflammatory M2-polarized macrophages in OA model; moreover, the imbalance of M1/M2 macrophages is closely linked to OA progression (7, 12, 23). Notably, both vitro and vivo studies reveal that reprogramming macrophages from M1 subtype to M2 subtype can attenuate the progression of OA, supporting this approach as a new promising therapeutic strategy (22, 24, 25). In OA, macrophages become activated and polarize to pro-inflammatory M1 phenotype that can release abundant pro-inflammatory cytokines to generate an inflammatory environment to exacerbate cartilage degeneration (26, 27). Similar with previous study (21), this study confirmed that carnosic acid obviously inhibited macrophage M1 polarization and inflammation, and further promoted M2-like macrophage polarization. Importantly, carnosic acid shifted synovial macrophage polarization from M1 to M2 phenotype, and attenuated inflammatory response in OA mice. Thus, carnosic acid may alleviated the progression of OA by reprogramming macrophage polarization-mediated inflammation. Intriguingly, the previous data highlighted that carnosic acid might attenuate collagen-induced rheumatoid arthritis by ameliorating inflammation and joint destruction (19).

Recently, convincing evidence has revealed that the cross-talk interactions between macrophages and chondrocytes play important roles in the initiation and progression of OA (11, 22, 28, 29). Activated M1 macrophages will release abundant pro-inflammatory cytokines to generate an inflammatory environment to affect chondrocyte injury and inflammatory response, thereby exacerbating articular cartilage degeneration in OA (11, 14, 28). In this study, carnosic acid inhibited M1 macrophage-mediated releases of TNF-α, IL-1β and IL-6. Intriguingly, co-culture with M1 macrophage conditioned medium increased inflammatory mediator and cytokine levels in chondrocytes. The typical inflammatory cytokine IL-6, TNFα and IL-1β represent three highly inflammatory cytokines in OA and can be released from chondrocytes and macrophages; these cytokines may promote destruction and degradation of articular cartilage (9, 30). Thus, carnosic acid may inhibit M1 macrophages-evoked chondrocyte inflammation.

Next, similar with previous study (11), our data confirmed that M1 macrophage conditioned medium induced chondrocyte apoptosis. Importantly, M1 macrophages enhanced oxidative injury of chondrocytes. Recent study has revealed the important roles of oxidative stress in the progression of OA (8, 9). Oxidative stress is a common consequence resulting from excessive generation of ROS that will weaken the cellular antioxidant defense system. Several studies have confirmed that the excessive oxidative stress status in chondrocytes may be related to the pathogenesis of OA (8, 9). Currently, reducing oxidative stress may represent a promising therapeutic strategy for OA (9, 14). In this study, carnosic acid attenuated M1 macrophage-evoked oxidative injury in chondrocytes. Moreover, high oxidative stress levels in OA mice were also abrogated by carnosic acid. Notably, a previous study confirmed that carnosic acid could attenuate gastric ulceration by inhibiting oxidative stress and inflammation (17). Moreover, carnosic acid combined with methotrexate may effectively alleviate the progression of adjuvant arthritis by inhibiting inflammation and oxidative stress (31). Furthermore, a previous study also confirmed that carnosic acid attenuated receptor activator of NF-κB ligand (RANKL)-induced oxidative stress injury in osteoclasts and osteoporosis progression, indicating its protective efficacy in osteoporosis (32).

Analogously with a previous study (29), this study revealed that M1 macrophages might enhance ECM degradation of chondrocytes by increasing catabolic metabolism-related MMP-3, MMP-13 levels and decreasing anabolic metabolism-related aggrecan and collagen II expression. Metabolism imbalance between catabolism and anabolism in ECM of articular cartilage represents a key event in the progression of OA (14, 33). During OA, exposure to inflammatory environment can disturb metabolic homeostasis of articular cartilage and increase catabolism by decreasing ECM synthesis-related aggrecan and collagen expression and increasing production of MMPs, such as MMP-3 (33). Notably, carnosic acid alleviated M1 macrophages-mediated metabolism disturbance towards catabolism. Importantly, carnosic acid also attenuated degeneration of articular cartilage and excessive catabolism-related protein expression in OA mice. Intriguingly, a previous study revealed that carnosic acid exhibited little cytotoxicity and could directly attenuate IL-1β-induced ECM metabolic imbalance of chondrocytes, supporting its protective potential in degradation of cartilage in OA (34). Thus, these data suggest that carnosic acid may alleviate degradation of cartilage in OA by indirectly affecting macrophage polarization-evoked and directly affecting chondrocyte metabolism imbalance between catabolism and anabolism.

Further mechanistic assay highlighted that carnosic acid enhanced activation of the Nrf2 pathway, thereby inhibiting activation of the NF‐κB signaling in OA. A growing body of evidence highlights that Nrf2 is a key transcription factor involved in multiple physiological and pathological processes, such as cartilage homeostasis. Activation of Nrf2 can interact with HO-1 to exert anti-inflammatory and anti-oxidative stress effects by suppressing activation of the NF‐κB signaling (35, 36). Moreover, emerging data suggest activation of the Nrf2 pathway may contribute to anti-inflammatory polarization of macrophages, representing a novel therapeutic approach for OA (10, 35, 37). Furthermore, knockdown of Nrf2 exacerbates oxidative stress and leads to more severe OA (8). Intriguingly, a substantial body of evidence reveals that inhibiting the NF‐κB signaling by Nrf2 can slow OA progression by suppressing apoptosis, inflammation and ECM degradation (36, 38). Intriguingly, a previous finding corroborated that carnosic acid may alleviate RANKL-induced oxidative stress and osteoclastogenesis by activating Nrf2 while suppressing the NF‐κB pathway (32). Consequently, these data indicate that the Nrf2/NF‐κB axis may account for the protective efficacy of carnosic acid in the progression of OA.

## 5 Conclusion

The current findings highlighted that carnosic acid inhibited macrophage M1 polarization and their polarization-induced chondrocyte oxidative injury, inflammation and ECM metabolism disorders towards catabolism by regulating the Nrf2/NF‐κB axis. Importantly, carnosic acid alleviated cartilage oxidative injury, macrophage pro-inflammatory polarization, and cartilage degeneration in OA mice. Thus, this study first reveals that carnosic acid may alleviate the progression of OA by inhibiting oxidative injury, macrophage polarization-evoked inflammation and ECM metabolism imbalance. Therefore, this study may provide new evidence regarding how carnosic acid attenuates the progression of OA, supporting its potential as a therapeutic agent for OA. However, whether carnosic acid can alleviate the pain/functional outcomes (such as gait, weight-bearing, and mechanical sensitivity) in the OA model. Does carnosic acid have better therapeutic efficacy than other Nrf2 agonists or macrophage-modulating agents in OA? These questions will be explored in the future.

## Data Availability

The raw data supporting the conclusions of this article will be made available by the authors, without undue reservation.

## Supplementary material

Supplementary Figure 1 Carnosic acid alleviates severity of OA mice. OA mice were administrated with 5, 10, 20 and 50 mg/kg of carnosic acid for 8 weeks. Then, the Safranin O staining was carried out. The OARSI scores were analyzed. ^***^*P* < 0.001 vs. sham group, ^###^*P* < 0.001 vs. OA groups.
