# Supplementary figures and images for: Administration with carnosic acid alleviates the development of osteoarthritis by attenuating macrophage polarization-mediated inflammation and cartilage oxidative damage and degradation via regulating Nrf2/NF-kB axis
Source: Front Immunol. 2026 Feb 10;17:1710302. doi: 10.3389/fimmu.2026.1710302 (PMC12929504; doi:10.3389/fimmu.2026.1710302)

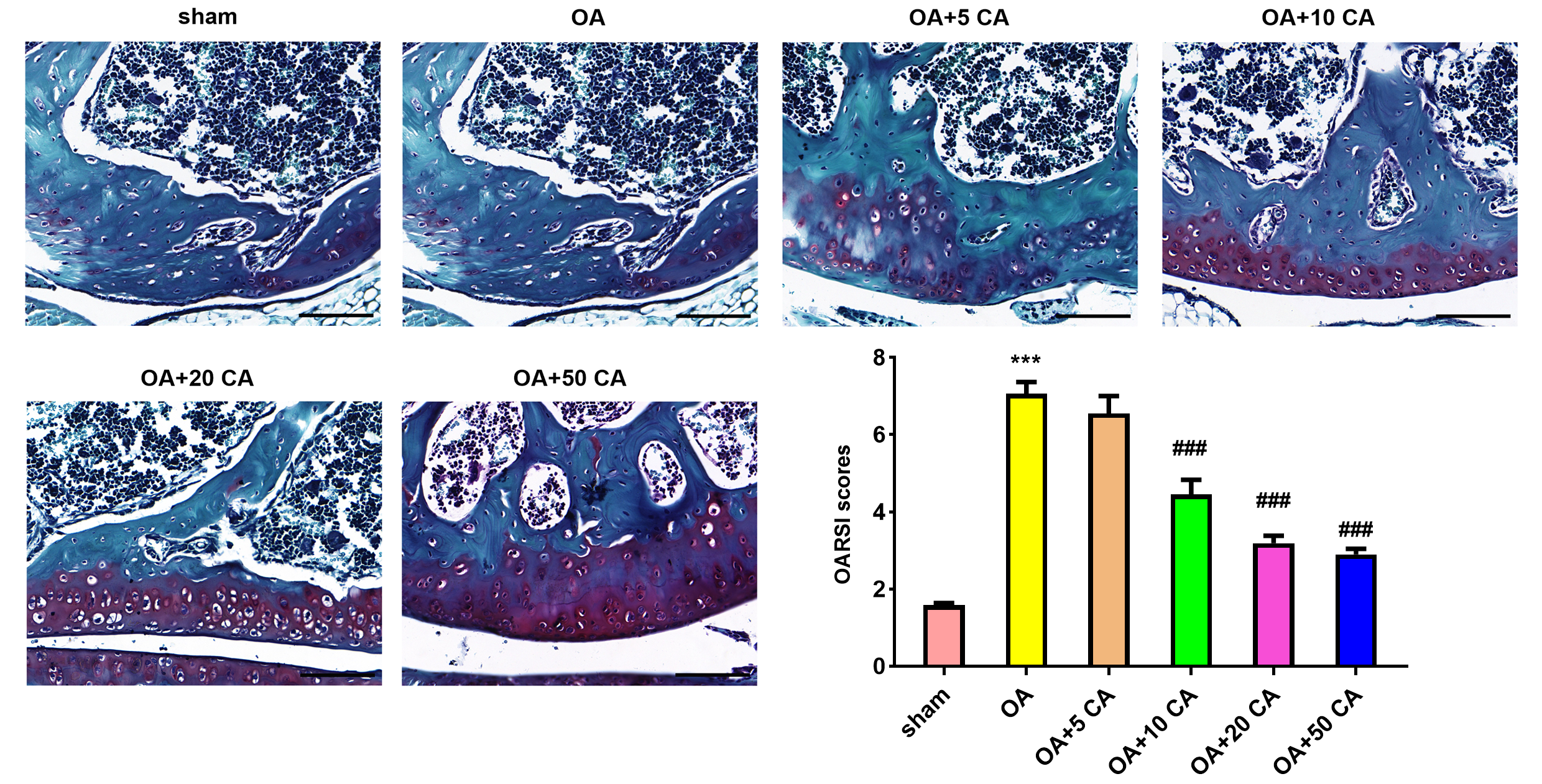

Supplement: Supplementary Figure 1 — Carnosic acid alleviates severity of OA mice. OA mice were administrated with 5, 10, 20 and 50 mg/kg of carnosic acid for 8 weeks. Then, the Safranin O staining was carried out. The OARSI scores were analyzed. ***P < 0.001 vs. sham group, ###P < 0.001 vs. OA groups. [file Image1.tif]
