# Supplementary material for: Administration with carnosic acid alleviates the development of osteoarthritis by attenuating macrophage polarization-mediated inflammation and cartilage oxidative damage and degradation via regulating Nrf2/NF-kB axis
Source: Front Immunol. 2026 Feb 10;17:1710302. doi: 10.3389/fimmu.2026.1710302 (PMC12929504; doi:10.3389/fimmu.2026.1710302)
Supplement: Supplementary file 3 [file Table1.docx]

Supplementary Table 1. Antibody information

| Name | Supplier | Catalog numbers | Dilution ratios | Application |
| --- | --- | --- | --- | --- |
| CD206 | Abcam | ab300621 | 1:50 | immunofluorescence |
| CD86 | Abcam | ab119857 | 1:100 | immunofluorescence |
| collagen II | Santa Cruz Biotechnology | sc-52658 | 1:100 | immunofluorescence |
| collagen II | Santa Cruz Biotechnology | sc-52658 | 1:1000 | western |
| aggrecan | Abcam | ab315486 | 1:1000 | western |
| ADAMTS5 | Abcam | ab41037 | 1:250 | western |
| Nrf2 | Abcam | ab313825 | 1:3000 | western |
| HO-1 | Abcam | ab68477 | 1:10000 | western |
| p-p65 NF-κB | Abcam | ab76302 | 1:1000 | western |
| p65 NF-κB | Abcam | ab32536 | 1:50000 | western |
| Bax | Abcam | ab32503 | 1:5000 | western |
| Bcl-2 | Abcam | ab182858 | 1:2000 | western |
| LaminB | Abcam | ab133741 | 1:6000 | western |
| β-actin | Abcam | ab8227 | 1:3000 | western |
