# Supplementary material for: Administration with carnosic acid alleviates the development of osteoarthritis by attenuating macrophage polarization-mediated inflammation and cartilage oxidative damage and degradation via regulating Nrf2/NF-kB axis
Source: Front Immunol. 2026 Feb 10;17:1710302. doi: 10.3389/fimmu.2026.1710302 (PMC12929504; doi:10.3389/fimmu.2026.1710302)
Supplement: Supplementary file 4 [file Table2.docx]

Table 2 Primer sequences of qRT-PCR

| Name | Primer sequences (5’-3’) |
| --- | --- |
| CD86 | sense, 5’-TCACCCGAAACCTAAGAAGATG-3’  anti-sense, 5’-AGAGAGAGGCTGTTGGAGATA-3’ |
| IL-1β | sense, 5’-ATGGGCAACCACTTACCTATTT-3’  anti-sense, 5’-GTTCTAGAGAGTGCTGCCTAATG-3’ |
| TNF-α | sense, 5’-GCCTCCCTCTCATCAGTTCTA-3’  anti-sense, 5’-CCATCTCATCCCATGCCTAAC-3’ |
| collagen II | sense, 5’-TCACCCGAAACCTAAGAAGATG-3’  anti-sense, 5’-AGAGAGAGGCTGTTGGAGATA-3’ |
| IL-6 | sense, 5’-ATTCCTCTGTGCCACCTTTAC-3’  anti-sense, 5’-GGTCAGCACCACCATCTTATT-3’ |
| iNOS | sense, 5’-GGAATCTTGGAGCGAGTTGT-3’  anti-sense, 5’-CCTCTTGTCTTTGACCCAGTAG-3’ |
| CD206 (Mrc1) | sense, 5’-GGCGAGCATCAAGAGTAAAGA-3’  anti-sense, 5’-CATAGGTCAGTCCCAACCAAA-3’ |
| CD163 | sense, 5’-CAGACTGGTTGGAGGAGAAATC-3’  anti-sense, 5’-CAGCTTCCAGAGACAAGTCAA-3’ |
| IL-10 | sense, 5’-TTGAATTCCCTGGGTGAGAAG-3’  anti-sense, 5’-TCCACTGCCTTGCTCTTATTT-3’ |
| aggrecan | sense, 5’-GTGGAGAGTCTTCTGGCATTAC-3’  anti-sense, 5’-CACTGAGTTCCACAGATCCTAAC-3’ |
| ADAMTS5 | sense, 5’-CTCGATCCCTAGCTGTCTTTG-3’  anti-sense, 5’-CAGGAGTGGCTTTAGAGTGTAG-3’ |
| MMP-3 | sense, 5’-GGACCAGGGATTAATGGAGATG-3’  anti-sense, 5’-TGAGCAGCAACCAGGAATAG-3’ |
| MMP-13 | sense, 5’-GACACAGCAAGCCAGAATAAAG-3’  anti-sense, 5’-GGAAAGCAGAGAGGGATTAACA-3’ |
| β-actin | sense, 5’-GAGGTATCCTGACCCTGAAGTA-3’  anti-sense, 5’-CACACGCAGCTCATTGTAGA-3’ |
